# RNA sequencing for global gene expression associated with muscle growth in a single male modern broiler line compared to a foundational Barred Plymouth Rock chicken line

**DOI:** 10.1186/s12864-016-3471-y

**Published:** 2017-01-13

**Authors:** Byung-Whi Kong, Nicholas Hudson, Dongwon Seo, Seok Lee, Bhuwan Khatri, Kentu Lassiter, Devin Cook, Alissa Piekarski, Sami Dridi, Nicholas Anthony, Walter Bottje

**Affiliations:** 1 Department of Poultry Science, Center of Excellence for Poultry Science, University of Arkansas, Fayetteville, Arkansas USA; 2 School of Agriculture and Food Science, University of Queensland, Gatton, Australia

## Abstract

**Background:**

Modern broiler chickens exhibit very rapid growth and high feed efficiency compared to unselected chicken breeds. The improved production efficiency in modern broiler chickens was achieved by the intensive genetic selection for meat production. This study was designed to investigate the genetic alterations accumulated in modern broiler breeder lines during selective breeding conducted over several decades.

**Methods:**

To identify genes important in determining muscle growth and feed efficiency in broilers, RNA sequencing (RNAseq) was conducted with breast muscle in modern pedigree male (PeM) broilers (*n* = 6 per group), and with an unselected foundation broiler line (Barred Plymouth Rock; BPR). The RNAseq analysis was carried out using Ilumina Hiseq (2 x 100 bp paired end read) and raw reads were assembled with the *galgal4* reference chicken genome. With normalized RPM values, genes showing >10 average read counts were chosen and genes showing <0.05 p-value and >1.3 fold change were considered as differentially expressed (DE) between PeM and BPR. DE genes were subjected to Ingenuity Pathway Analysis (IPA) for bioinformatic functional interpretation.

**Results:**

The results indicate that 2,464 DE genes were identified in the comparison between PeM and BPR. Interestingly, the expression of genes encoding mitochondrial proteins in chicken are significantly biased towards the BPR group, suggesting a lowered mitochondrial content in PeM chicken muscles compared to BPR chicken. This result is inconsistent with more slow muscle fibers bearing a lower mitochondrial content in the PeM. The molecular, cellular and physiological functions of DE genes in the comparison between PeM and BPR include organismal injury, carbohydrate metabolism, cell growth/proliferation, and skeletal muscle system development, indicating that cellular mechanisms in modern broiler lines are tightly associated with rapid growth and differential muscle fiber contents compared to the unselected BPR line. Particularly, PDGF (platelet derived growth factor) signaling and NFE2L2 (nuclear factor, erythroid 2-like 2; also known as NRF2) mediated oxidative stress response pathways appear to be activated in modern broiler compared to the foundational BPR line. Upstream and network analyses revealed that the MSTN (myostatin) –FST (follistatin) interactions and inhibition of AR (androgen receptor) were predicted to be effective regulatory factors for DE genes in modern broiler line. PRKAG3 (protein kinase, AMP-activated, gamma 3 non-catalytic subunit) and LIPE (lipase E) are predicted as core regulatory factors for myogenic development, nutrient and lipid metabolism.

**Conclusion:**

The highly upregulated genes in PeM may represent phenotypes of subclinical myopathy commonly observed in the commercial broiler breast tissue, that can lead to muscle hardening, named as woody breast. By investigating global gene expression in a highly selected pedigree broiler line and a foundational breed (Barred Plymouth Rock), the results provide insight into cellular mechanisms that regulate muscle growth, fiber composition and feed efficiency.

**Electronic supplementary material:**

The online version of this article (doi:10.1186/s12864-016-3471-y) contains supplementary material, which is available to authorized users.

## Background

Production efficiency in animal agriculture continues to be of vital importance to meet the protein nutrition needs of an ever increasing global human population. Genetically selected modern broiler chickens acquired highly improved production efficiency through rapid growth and high feed efficiency compared to unselected chicken breeds. Thus, understanding of mechanisms regulating rapid muscle growth and high feed efficiency may play an increasingly important role in developing more efficient, and therefore sustainable, animal production systems [1].

A global gene expression study using cDNA microarray assay was conducted on breast muscle phenotyped by high and low production efficiency in male pedigree broiler breeder chickens [2–4]. Additional global gene expression studies have been performed with breast muscle tissue using RNAseq in commercial broilers [5] and duodenal tissue using RNAseq in broilers selected for low and high residual feed intake (RFI) [6]. Several global expression studies showed that production efficiency may be associated with various cellular mechanisms including mitochondrial oxidative stress, inflammatory response, protein degradation, stress responses, growth hormone signaling, cell cycle, apoptosis and fatty acid transportation. A recent study on the transcriptome analyses of breast muscles in modern pedigree broiler chicken and legacy chicken lines reported that the transcriptional profile of differentially enriched genes on day 6 and day 21 in modern broiler muscles includes genes for enhanced myogenic growth and differentiation [7].

The Barred Plymouth Rock (BPR) chicken, with its characteristic pattern of alternating white and black bars of feather pigmentation were developed by crosses involving the Black Java, Black Cochin, and Dominique breeds in America during the mid-19th century [8] and is a foundational or heritage breed of the modern commercial broilers. The BPR breed, originally developed for the dual purposes of both meat and egg production, has much slower growth rate (589 g empty body weight at 42 days) compared to commercial broilers (2276 g at 42 days) [9].

With distinct differences in growth characteristics, the objective of the present study was to conduct global gene expression analyses on breast muscle tissue obtained from modern pedigree male (PeM) broilers (rapid growth and muscle development) compared with the foundational BPR chickens (slow growth and lower efficiency) [9]. Recent popular RNA-sequencing method using massively parallel nucleotide sequencing techniques were used for the comparison of global gene expression in this study. From this comparison, DE genes showing greater differences between the two groups may provide more definitive insights into cellular regulatory mechanisms in muscle growth and feed efficiency.

## Methods

### Ethics Statement

The present study was conducted in accordance with the recommendations in the guide for the care and use of laboratory animals of the National Institutes of Health. All procedures for animal care complied with the University of Arkansas Institutional Animal Care and Use Committee (IACUC): Protocol #14012.

### Animals, breast muscle tissue and RNA extraction

Breast muscle tissue analyzed in this study were obtained from immature (≤8 weeks old) pedigree broiler males (PeM) and BPR. The PeM samples had been obtained previously [2] and from an inbred male line highly selected for growth and feed efficiency [10]. Briefly, immature PeM and BPR chickens (<8 weeks old, *n* = 6 per breed) were killed by an overdose of sodium pentobarbital (i.v.) and breast muscle tissue obtained and flash frozen in liquid nitrogen. RNA was extracted from breast muscle using TRIzol reagent (Invitrogen Life Technologies, Thermo-Fisher Scientific, Carlsbad, CA) following the manufacturer’s instructions. After the first RNA extraction, the RNA samples were treated with DNase I (New England Biolabs Inc., Ipswich, MA) and extracted again with TRIzol reagent. The RNA quality was then assessed by Agilent 2200 TapeStation instrument (Santa Clara, CA). The RNA samples were used for RNA sequencing (RNAseq) analysis.

### RNAseq and data analysis

RNAseq library preparation for individual samples with barcoding and Illumina sequencing were performed by the Research Technology Support Facility at Michigan State University (East Lansing, MI). Illumina HiSeq system 2x100 bp paired end read technology was used for RNAseq. The RNA sequence FASTA files were mapped to the chicken genome *(galgal4)* retrieved from Ensembl using CLC Genomics Workbench software (Qiagen, Valencia, CA; licensed to CSIRO) following the software manual (http://resources.qiagenbioinformatics.com/manuals/clcgenomicsworkbench/752/User_Manual.pdf; http://www.clcbio.com/wp-content/uploads/2012/08/RNA-Seq_analysis_part_I.pdf) and the RNAseq analytical pipeline recommended in [11]. Briefly, raw reads were processed by reads’ quality control and adapter trimming with default setting. Filtered reads were subjected to reference based-RNAseq pipeline of CLC software with default settings. The EMBL annotation files of reference chicken genome were downloaded from Ensembl (ftp://ftp.ensembl.org/pub/release-87/embl/gallus_gallus/) and tracks for sequence and genes were generated with track conversion. The parameters of initial RNAseq mapping are as followings: Use reference genome annotated with genes and transcripts; Eukaryote origin; Map to gene region only; mapping options with 2 mismatch costs, 3 insertion costs, 3 deletion costs, 0.7 length fraction, 0.8 similarity fraction, both strand specific, 10 maximum number of hits for a read; total counts for expression value. Total mapped counts were transformed to log_2_ values of the number of reads per million (RPM) to stabilize the variance and then a further quantile normalization was performed [11]. Normalized values were subjected to further statistical analyses performed by JMP Genomics 6 (SAS Institute Inc., Cary, NC). Genes with less than 10 average raw read counts in both comparison groups were removed and not considered for further analyses. The t-statistics was used to compare between PeM and BPR. Genes showing >1.3 fold differences and <0.05 p-value in the comparison between PeM and BPR were considered differentially expressed (DE) genes. The p-value correction (FDR calculation) by multiple tests was not applied in this study since we used a less stringent approach on a gene by gene basis, and this allowed us to import much more informative lists and assess the genome-wide data using various alternative approaches, such as the hyper-geometric stats used by functional enrichments for pathway analyses, the co-expression patterns in the hierarchical heatmaps, and the binomial distributions in the mitoproteome plot [12, 13].

### Mitoproteome analysis

The complete human mitoproteome was downloaded from http://mitominer.mrc-mbu.cam.ac.uk/release-3.1/begin.do generating a list of 1,046 nuclear and mitochondrial genes encoding mitochondrial proteins. Protein names were converted to gene names and 699 genes matched the chicken RNAseq data in this study. The expression of mitoproteome genes was then plotted in MA format, and all those with higher values in the PeM group were color coded red and those with lower values in the PeM color coded blue. The skew in distribution away from the null expectation of 50:50 was quantified by binomial statistics. This approach formalizes the extent to which the mitoproteome data is biased to one or the other of the two groups.

### Hierarchical clustering analysis

The genes with greatest DE (20 up- and 20 down-regulated) between PeM and BPR were subjected to hierarchical cluster analysis using the JMP Genomics program. We imported the matrix with as many columns as birds (12) and as many rows as genes (40), where each cell contains the log_2_ transformed fold change value for that gene and individual into JMP Genomics program, normalizing on rows. We applied hierarchical clustering on both rows and columns and exported the resulting dendrogram as an image file.

### Pathway analyses

Ingenuity Pathway Analysis (IPA; Qiagen, Valencia, CA; http://www.ingenuity.com) software was used for functional annotation, canonical pathway analysis, upstream analysis and network discovery. Because IPA is based on the bioinformatics in humans, functionalities for differentially expressed genes in our chicken datasets are based primarily on mammalian biological mechanisms. As the investment in biomedical research biases the functional annotations towards human disease, we have attempted to draw plausible conclusions based on avian based literature.

### Quantitative RT-PCR

One microgram of total RNA was obtained from 6 muscle samples each for PeM and BPR for general validation of the RNAseq results and for specific confirmation of the top 10 genes, respectively. RNA was converted into cDNA with qScript™ cDNA SuperMix (Quanta Biosciences, Gaithersburg, MD) following the manufacturer’s instructions. The cDNA samples were diluted in a 1:10 ratio and a portion (2 μL) of the cDNA was subjected to a real-time quantitative PCR (qPCR) reaction using an ABI prism 7500HT system (ThermoFisher Scientific, Waltham, MA) with PowerUp™ SYBR® Green Master Mix (ThermoFisher Scientific, Waltham, MA). The specific oligonucleotide primers were designed by the PRIMER3 program (http://frodo.wi.mit.edu) and the list of primers were listed in Additional file 1. The conditions of real-time qPCR amplification were 1 cycle at 95 °C for 2 min, 40 cycles at 95 °C for 30 s, 65 °C for 30 s. The chicken glyceraldehyde 3-phosphate dehydrogenase (GAPDH) gene was used as the internal control. Dissociation curves were performed at the end of amplification for validating data quality. All qPCR reactions were performed 3 times and the values of average cycle threshold (Ct) were determined for each sample, and 2^−ΔΔCt^ values for the comparison of PeM and BPR were used for relative quantification by fold-change and statistical significance.

## Results and discussion

### RNAseq results

**Fig. 1:**
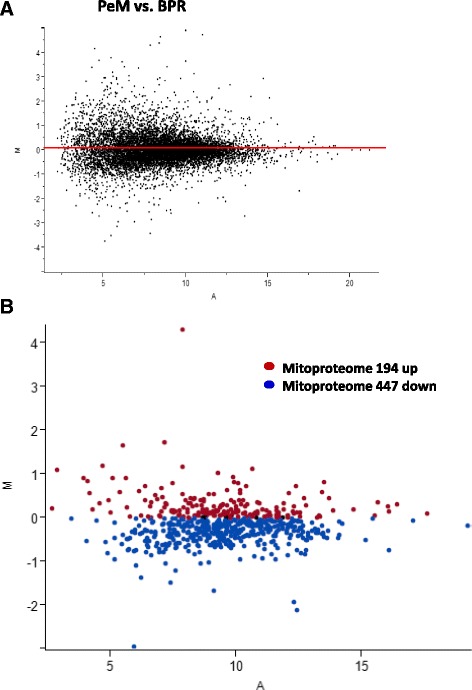
MA plots and number of DE genes. (**a**) MA plot for the comparison of PeM and BPR. (**b**) MA plot for mitoproteome genes of PeM vs. BPR. In (**a**) and (**b**), y and x axis represent the log ratio of differential expression and the average expression, respectively, for each gene in groups of PeM and BPR

**Fig. 2:**
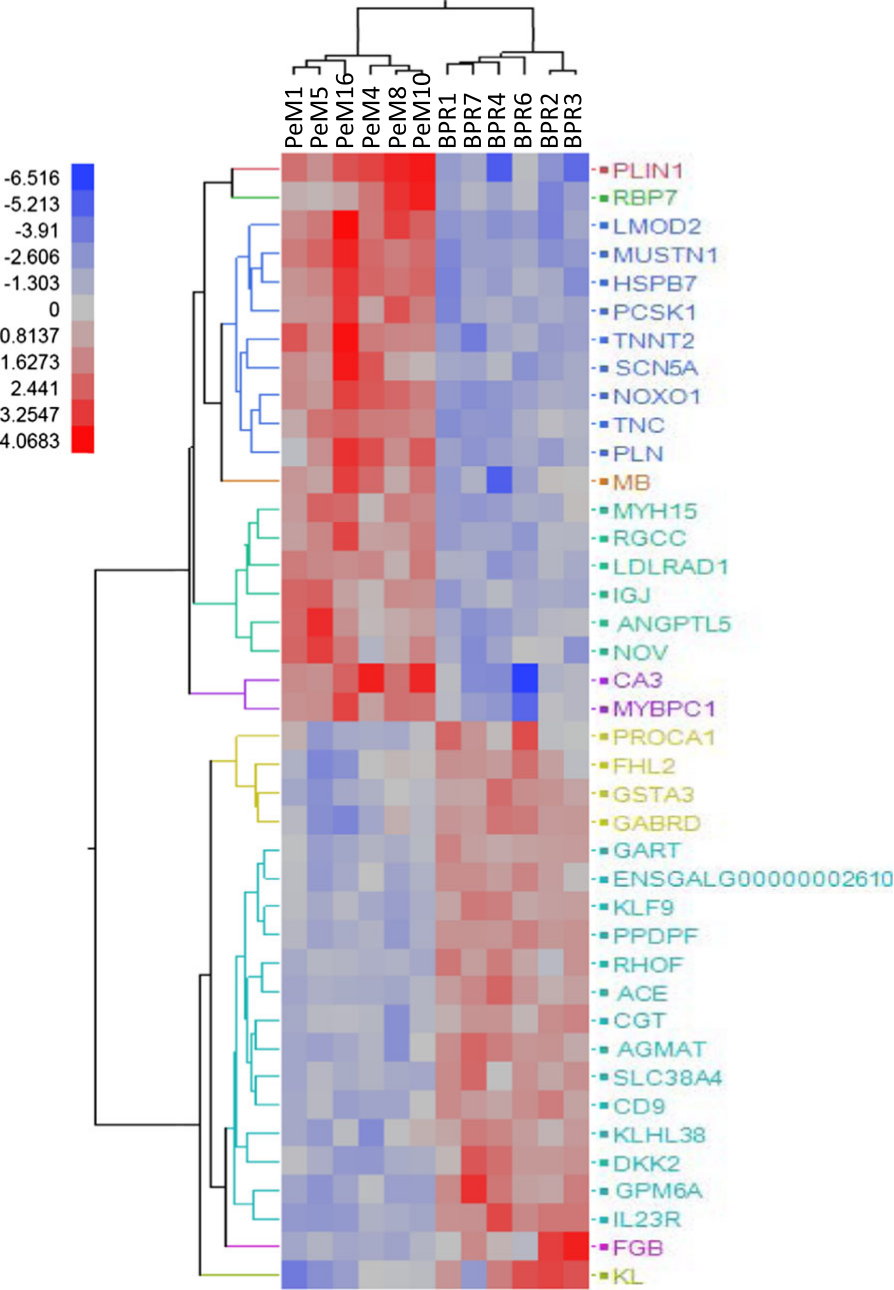
A hierarchically clustered heatmap showing the expression patterns of the 40 most extreme DE mRNA. Red and blue represent up- and downregulated expression in PeM, respectively. Color density indicating levels of fold change was displayed

Twelve RNAseq libraries were constructed using RNA samples of breast muscles from 6 chickens each for PeM and BPR and sequenced with 2x 100 bp paired end reads on 4 lanes. Totally ~800 million 100 bp sequence reads were obtained with an average of ~67 million reads per sample and ~80% of sequence reads were mapped to the chicken reference genome (*galgal4*). After quantile normalization with log_2_ transformation of the number of reads per million (RPM) and filtering out very low expression genes (raw read counts <10), 9,263 genes remained and the MA plot showed that up- and down-regulated genes were evenly distributed in the comparison between PeM and BPR (Fig. 1a). Finally, 2,464 DE genes showing <0.05 p-value and >1.3 fold change were identified in the comparison between PeM and BPR (Additional File 2). To validate RNAseq results, a subset of 20 genes selected from the DE list were subjected to qPCR. These were genes associated with muscle structures [slow twitch subunits (MYH15, TPM3, MYOZ2, TNNI1, MYBPC1), slow muscle associated proteins (MB, CA3), muscle fat content (PLN, FABP4), muscle growth/differentiation (MSTN)], mitochondrial biogenesis and proteins (PPARGC1A, PPARGC1B, CKMT1A, NOXO1), and five out of the top 10 downregulated genes (IL23R, GPM6A, PPDPF, ACE, CD9). Results indicated that expression levels for 16 genes out of 20 tested were well-matched between RNAseq and qPCR analyses in terms of the direction and magnitude of change (Table 1). Four genes were not matched between RNAseq and qPCR for reasons that are not clear to us, but one possibility relates to the fact that the two methods use quite different normalization approaches. Those transcripts where the two methods make similar DE predictions can be considered the most robust of our reported data. Additionally, hierarchical clustering showed clear discrimination the 12 birds into the correct group of origin (Fig. 2).

**Table 1:** Comparison of fold changes between RNAseq and qPCR

	PeM vs. BPR	
Gene symbol	RNAseq*	qPCR*
MYH15	9.2	5.1
TPM3	2.3	2.2
MYOZ2	5.9	4.4
TNNI1	5.7	24.7
MSTN	2.2	1.4
PLN**	*13.8*	*−2.4*
PPARGC1A	−2.2	−1.5
PPARGC1B	−2.3	−1.7
FABP4	6.5	5.0
CKMT1A	1.6	1.3
MB	10.9	10.3
LMOD2**	*32.2*	*−1.1*
CA3	30.3	4.6
NOXO1	18.1	1.2
MYBPC1	12.6	10.8
IL23R**	*−13.4*	*1.2*
GPM6A	−11.4	−1.7
PPDPF	−6.5	−5.3
ACE**	*−6.0*	*1.1*
CD9	−5.9	−4.5

* Values denote linear fold changes

** Inconsistent fold change between RNAseq and qPCR were italicized

Of the 699 genes that were detected that encode proteins localized to the mitochondrion, 194 and 447 were numerically higher and lower, respectively, in PeM compared to BPR, while 58 genes were not changed (Fig. 1b). This is a very significant skew (binomial P value < 0.000001) implying the PeM birds have a lowered mitochondrial content. Recently, a breast muscle transcriptomic analyses using RNAseq method was reported on the comparison between the broiler pedigree Ross and a legacy Illinois chicken line [7]. When raw sequencing data, which is available on Gene Expression Omnibus (GEO), were reanalyzed for gene expression, the 608 genes encoding proteins localized to the mitochondrion were differentially expressed as 251 and 357 were up- and downregulated, respectively, in the 21 day old Ross chickens compared to Illinois chickens (data not shown; Seo et al., manuscript in preparation). Similar findings of these two independent studies showing a lower number of upregulated genes encoding mitochondrial proteins in modern broiler compared to unselected chicken breeds may strongly support reduced numbers of mitochondrial content in modern breeds selected for growth and feed efficiency.

This mitochondrial content finding is in line with the economic physiological design argument proposed by Hudson [14] in the context of production animal feed efficiency. This argument proposes that an economically designed feed converter should have enough, but not too much, fuel burning capacity. This is because spare capacity bears costs of construction (mitochondrial biogenesis), maintenance (trans-membrane proton gradient) and load (space that could be used for other organelles) that should be avoided if they are unnecessary.

### Hierarchical clustering on extreme DE

The top 20 up- and 20 downregulated genes were subjected to hierarchical clustering analysis (Fig. 2). The results clearly discriminate the 12 birds into the correct group of origin. Functional groups contained in the 20 upregulated genes in PeM include cytoskeleton and muscle contraction [LMOD2 (leiomodin 2, cardiac), CA3 (carbonic anhydrase III), MUSTN1 (musculoskeletal, embryonic nuclear protein 1), TNNT2 (troponin T type 2, cardiac), MYBPC1 (myosin binding protein C, slow type), MB (myoglobin), MYH15 (myosin heavy chain 15)], extracellular matrix [or secreting proteins; TNC (tenascin C), ANGPTL5 (angiopoietin-like 5), NOV (nephroblastoma overexpressed), IGJ (joining chain of multimeric IgA and IgM)], transporter [PLN (phospholamban), MB], protease [PCSK1 (proprotein convertase subtilisin/kexin type 1)], and ion channel [SCN5A (sodium channel, voltage gated, type V alpha subunit)]. Functional groups contained in the 20 downregulated genes include transcription factors [FHL2 (four and a half LIM domains 2), KLF9 (Kruppel-like factor 9)], calcium signaling [PROCA1 (protein interacting with cyclin A1), GPM6A (glycoprotein M6A)], synthetic enzymes [GART (phosphoribosylglycinamide formyltransferase), CGT (UDP glycosyltransferase 8), RHOF (ras homolog family member F), GSTA3 (glutathione S-transferase alpha 3)], protein ubiquitination [KLHL38 (kelch-like family member 38)], membrane bound [SLC38A4 (solute carrier family 38, member 4), CD9 (CD9 molecule), ACE (angiotensin I converting enzyme), GPM6A, IL23R (interleukin 23 receptor)], and secreting protein [DKK2 (dickkopf WNT signaling pathway inhibitor 2), KL (klotho)]. More details on the function of a narrower list for the 10 most DE genes are described below.

### The 10 most DE genes

The full name of the 10 most DE genes between PeM and BPR muscle and their functional characteristics are shown in Tables 2 and 3. Interestingly, highly upregulated genes in PeM muscle are closely associated with expression of gene and phenotypes of subclinical myopathy commonly observed in the commercial broiler breast tissue, as discussed below. Five of the 10 most upregulated genes (LMOD2, CA3, MUSTN1, TNNT2, and MYBPC1) in PeM are mainly expressed in skeletal or cardiac muscles (Tables 2 and 3), particularly in slow twitch fibers (type 1), except for MUSTN1 which is not expressed in slow twitch fibers. This observation indicates that breast muscle in PeM chickens could have higher slow fiber composition compared to BPR muscle. When considering the mitoproteome and mitochondrial contents discussed earlier, the possible higher slow fiber composition in PeM is counter to the lowered gene expression for mitoproteome, which are typically associated with fast twitch muscle fibers. Generally, slow-twitch or oxidative fibers have high mitochondrial content, slow contraction rates, increased reliance on oxidative phosphorylation (OXPHOS), high resistance to fatigue, and high representation in postural muscles. In contrast, fast-twitch or glycolytic fibers have lower mitochondrial content, rapid contractions, decreased reliance on OXPHOS, low resistance to fatigue, and high representation in muscle groups used for directional movement [15]. In general, we know that chickens have a white breast muscle with a very high preponderance of fast twitch glycolytic fibers bearing a low mitochondrial content (e.g. [16]). Based on the DE genes encoding mitochondrial proteins (Fig. 1b), modern PeM breast muscles may contain an even higher content of fast twitch fibers compared to BPR muscle. However, the most upregulated genes showed the possibility of muscle composition, that contain higher gene expression for the slow muscle fiber composition in modern PeM compared to BPR. This indicates that chicken breast muscle mostly consists of fast fibers, more rapidly growing modern PeM broiler muscle contains increased slow fiber compositions, and the increased mass of slow fibers may be regulated independently of mitochondrial content and function. It also suggests that the mitochondria in avian muscle may function to regulate muscle mass growth by modulating oxidative stress, but may not regulate fiber composition. These results may represent a fundamentally new finding about muscle physiology in birds. In an earlier report by Mutryn et al. [17], wooden breast myopathy muscle (which in commercial broilers showed a dramatic upregulation of TNNI1, MB, MYBPC1, and MYOZ2 genes compared to unaffected muscle), suggested fiber type switching from fast- to slow type fibers. In addition to the 10 most upregulated genes, expression of MB (10.85 fold) and MYOZ2 (5.90 fold) were also highly upregulated in PeM compared to BPR (Additional file 2). Taken together, PeM muscles may contain increased slow fiber compositions compared to BPR.

**Table 2:** The 10 most differentially expressed genes in breast muscle tissue modern PeM broiler males compared to progenitor BPR broilers

FC	*p*-value	Symbol	Entrez gene name
Up-regulation			
48.16	6.4E-05	PLIN1	perilipin 1
32.22	9.2E-07	LMOD2	leiomodin 2 (cardiac)
30.27	7.9E-04	CA3	carbonic anhydrase III
26.72	1.7E-07	MUSTN1	musculoskeletal, embryonic nuclear protein 1
20.96	1.2E-05	TNNT2	troponin T type 2 (cardiac)
19.97	4.8E-06	HSPB7	heat shock 27 kDa protein family, member 7 (cardiovascular)
18.12	1.0E-06	NOXO1	NADPH oxidase organizer 1
13.83	8.1E-05	PLN	phospholamban
12.99	2.8E-05	PCSK1	proprotein convertase subtilisin/kexin type 1
12.55	9.2E-04	MYBPC1	myosin binding protein C, slow type
Down-regulation			
−13.36	9.6E-07	IL23R	interleukin 23 receptor
−11.39	4.2E-05	GPM6A	glycoprotein M6A
−8.81	1.5E-02	KL	klotho
−7.72	7.8E-05	AGMAT	agmatine ureohydrolase (agmatinase)
−7.72	1.1E-04	DKK2	dickkopf WNT signaling pathway inhibitor 2
−7.21	1.4E-03	GABRD	gamma-aminobutyric acid (GABA) A receptor, delta
−6.54	4.5E-03	FGB	fibrinogen beta chain
−6.49	1.1E-06	PPDPF	pancreatic progenitor cell differentiation and proliferation factor
−6.02	4.5E-06	ACE	angiotensin I converting enzyme
−5.93	3.6E-05	CD9	CD9 molecule

**Table 3:** Biological functions of the 10 most up- and down-regulated genes

Symbol	Functions
Up-regulation	
PLIN1	• Is a member of the PLIN family, which has a specific role in regulating lipolysis • Expresses only in adipose tissue, not in skeletal muscle in mammals [18] • Limits the activity of the rate-limiting lipase, adipose triglyceride (ATGL) in a basal state [67] • Induces hormone-sensitive lipases recruitment by its phosphorylation [68]
LMOD2	• Is a member of tropomodulin family which is an actin filament elongation protein • Is restricted to skeletal and cardiac muscle [69] • Functions to maintain thin filament lengths in the mature heart [70]
CA3	• Is a class of metalloenzymes that catalyze the reversible hydration of CO_2_ • Expresses at high levels in skeletal muscle and much lower levels in cardiac and smooth muscle [71] • Functions to generate both bicarbonate and hydrogen ions for maintenance of pH homeostasis
MUSTN1	• Expresses predominantly in the skeletal muscles and tendons in chicken and mammals [72] • Knock-down leads to the inhibition of myogenic fusion and differentiation [73] • Plays a role in muscle development
TNNT2	• Is a tropomyosin binding subunit of the troponin complex, which is located on the thin filament of striated muscles • Regulates muscle contraction in response to alterations in intracellular Ca^2+^ concentration [74]
HSPB7	• Is widely expressed throughout the body [75] • Prevent polyQ-induced toxicity in mammalian cells by suppressing aggregation and does not induce autophagy [76]
NOXO1	• Regulates NOX1 positively [77] • Functions in superoxide production [78]
PLN	• Is a major substrate for the cAMP-dependent protein kinase in cardiac muscle • Regulates sarcoplasmic reticulum calcium uptake which is mediated Ca^2+^-ATPase by phosphorylation • Functions in muscle contraction/relaxation mainly in cardiac muscle [79]
PCSK1	• Is a member of serine endopeptidase family • Cleaves amino acid residues and modulates precursor proteins • Involves in glucose and lipid metabolism [20, 21]
MYBPC1	• Is a member of the myosin-binding protein C family • Functions as a major myosin thick filament regulatory proteins [80] • Plays an important role in muscle contraction by recruiting muscle-type creatine kinase to myosin filaments [81]
Down-regulation	
IL23R	• Associates constitutively with Janus kinase 2 (JAK2), and also binds to transcription activator STAT3 in a ligand-dependent manner [82]
GPM6A	• Plays a role as a modulator for neurite outgrowth and spine formation [83] • Functions as a nerve growth factor-gated Ca^2+^ channel in neuronal differentiation [84]
KL	• Is a type-I membrane protein that is related to beta-glucosidases • Is an important FGF23 signaling cofactor [85] • Results in chronic renal failure by reduced production [86]
AGMAT	• Converts agmatine to putrescine in the pathway of arginine-agmatine-polyamine pathway • Functions in embryonic survival, growth, and development [87]
DKK2	• Antagonize Wnt signaling by binding to LRP5/6 and a single-transmembrane protein called Kreme [88] • Has a role in osteoblast differentiation into mineralized matrices [89]
GABRD	• Is a ligand-gated chloride channel • Functions in neurosteroid modulation [90]
FGB	• Is a blood-borne secreting glycoprotein • Has roles in cell adhesion and spreading, • Functions as mitogen [91]
PPDPF	• Is also known as exocrine differentiation and proliferation factor (EXDPF) • Expresses highly in the exocrine cell progenitors and differentiated cells of the developing pancreas in zebrafish [92]
ACE	• Involves in catalyzing the conversion of angiotensin I into a physiologically active peptide angiotensin II. • Plays a key role in the renin-angiotensin system • Expresses in skeletal muscle and is potent to generate skeletal muscle atrophy with angiotensin II and AT-1 receptor (AGTR1) functions [23]
CD9	• Is a member of the transmembrane 4 superfamily, also known as the tetraspanin family • Is a cell surface glycoprotein • Functions in differentiation, adhesion, and signal transduction [93]

Regarding upregulation of PLIN1 (perilipin 1) in PeM, mammalian PLIN1 is known to be expressed in adipose tissue, but not in skeletal muscle [18]. In this study, PLIN1 from each bird showed a very low expression level (range of 10 – 66 raw read counts) in BPR breast muscle while relatively higher expression (range of 260 – 1,700 raw read counts) was observed in PeM breast muscles. Possibly, these data indicate that a potentially new pathway related to hormone sensitive lipase is activated in the large muscle mass of modern broilers compared to the leaner mass in the unselected foundation BPR line. Additionally, increased expression of adipose genes showed that PeM muscles may retain increased mass of adipose tissues, where the wooden breast myopathy showed increased occurrence of irregular adipose tissue throughout the muscle [19]. Higher expression of NOXO1 (NADPH oxidase organizer 1) indicates that oxidative stress and scavenging antioxidants are closely associated with feed efficiency, which has been reported previously [2, 3, 5]. PCSK1 (proprotein convertase subtilisin/kexin type 1) is not restricted to the expression in muscle tissues, and its functions in glucose and lipid metabolism [20, 21] implies that PeM muscle has more active cellular pathways for energy metabolism. Active energy metabolism has been known to be closely connected to oxidative stress in muscle [22].

In contrast to the 10 most up-regulated genes, expression of the 10 most down-regulated genes in PeM muscles are not restricted to muscle, but are expressed in most tissues and organs (Tables 2 and 3). Of the 10 most down-regulated genes, six genes are classified to membrane bound channels and secreting factors [IL23R, KL, GPM6A, GABRD (gamma-aminobutyric acid (GABA) A receptor, delta), FGB (fibrinogen beta chain), PPDPF (pancreatic progenitor cell differentiation and proliferation factor) and CD9], indicating that PeM muscles may retain characteristics of lower amounts of secreting factors compared to BPR muscles. The expression of ACE, in combination with angiotensin II and AGTR1 (angiotensin II type 1 receptor), can produce muscle atrophy [23], indicating that down-regulation of ACE in PeM muscle may contribute to muscle hypertrophy compared to BPR chickens. Davis et al. [7] also observed elevated ACE in a heritage (Illinois) broiler compared to modern broiler (Ross) and hypothesized that this would play a role in lowering protein synthesis and muscle development in the slower growing Illinois broiler.

### Overview of IPA analysis

For more stringent pathway analyses, DE genes in muscle from PeM and BPR were subjected to biological functional analyses using Ingenuity Pathway Analysis (IPA, Qiagen, Valencia, CA) and the analytic settings were limited to publications on muscle tissue only. The summary of IPA analysis in diseases, molecular, cellular and physiological functions for DE genes are presented in Table 4. The most relevant biological functions in skeletal muscles comparison between PeM and BPR included organismal injury/abnormalities, carbohydrate metabolism, cellular growth/proliferation, small molecule biochemistry, abnormal morphology of muscle, angiogenesis of skeletal muscle, and myogenesis of skeletal muscles. Further functional analyses including major functions of DE genes, canonical pathways, biological functions, upstream analyses, and molecular networks that can influence muscle growth and feed efficiency are discussed below.

**Table 4:** Top diseases, molecular, cellular, and physiological functions for differentially expressed genes in PeM compared to BPR

Functions	*P*-Value	Molecules
Diseases and disorders		
Organismal Injury and Abnormalities	3.22E-02	CXCL1, EGR1, IL13RA2, NOX4
Molecular and Cellular Functions		
Carbohydrate Metabolism	2.19E-02	ACACB, CRAT, FABP3, GFPT1, NHLRC1, NRG1, NUAK1, PKNOX1, PPARD, PPARG, PRKAA1, PRKAB2, PRKCB, PRKCD, UCP3, UGP2
Cellular Growth and Proliferation	2.18E-02	ATM, DCN, MSTN, HEY1, MYC, MYF5, PITX2, PRNP
Small Molecule Biochemistry	2.19E-02	CRAT, NRG1, PKNOX1, PPARG, PRKAA1, PRKCD, UCP3
Physiological System Development and Function		
Skeletal and Muscular System Development and Function - Abnormal Morphology of muscle	1.89E-02	CALCRL, CHRNG, COL6A1, CRK, CXCL12, DES, DLG1, ELN, ESR2, FBN1, FN1, FST, HEY1, HLX, INPP5days, KLF2, LMO7, LOX, LPL, MB, MEOX2, MGP, MMP2, MSTN, MTM1, MYF5, NGFR, PITX2, PPARGC1A, SRPK3
- Angiogenesis of Skeletal Muscle	9.22E-03	CXCL12, MYH9, PPARGC1A, PPARGC1B, VEGFA
Embryonic Development - Myogenesis of Skeletal Muscle	1.25E-02	CAPN3, CSRP3, CXCL12, ELN, GPCPD1, HLX, MEOX2, MSC, MSTN, MYF5, MYH9, PITX2, PPARGC1A, PPARGC1B, RGS2, SIX1, SRPK3, SVIL, UTRN, VEGFA26

### Downregulation of mitochondrial function in PeM muscle

A total of 168 differentially expressed mitoproteome genes were independently analyzed by IPA to characterize functional alterations of mitochondrial energy expenditure and cellular respiration. As expected, mitochondrial functions including oxidative phosphorylation, and TCA cycle activity were downregulated in PeM muscles compared to BPR muscles (Additional file 3: Figure S1 and Additional file 4). Mitochondrial complex 2, 3, 4, and 5 were downregulated in PeM (with no change in complex 1) and the genes responsible for oxidative phosphorylation were decreased in PeM compared to BPR. Likewise, the responsible genes for TCA cycles were decreased in PeM compared to BPR (Additional file 4).

### Canonical Pathways

IPA analysis generated 380 canonical pathways with DE genes and most of them were associated with general signal transduction pathways which are not limited to muscle growth/mass or feed efficiency. Thus, the two most relevant pathways for rapid growth and greater mass of muscle (up-regulated PDGF signaling pathway; [24, 25]) and feed efficiency (Up-regulation of NFE2L2 mediated oxidative stress pathway; [5, 26]) are discussed below. Other potentially important canonical pathways will be discussed in future study.

#### Up-regulated PDGF signaling pathway

**Fig. 3:**
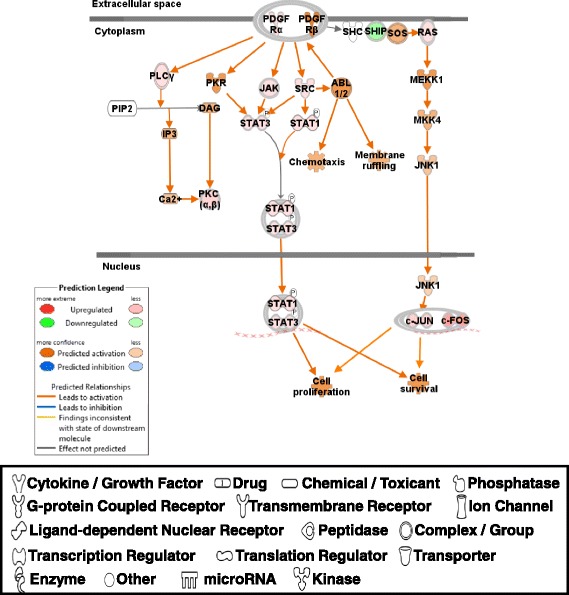
PDGF signaling pathway. Green, red, orange and blue represent down-regulation, up-regulation, predicted up-regulation and predicted down-regulation, respectively. Symbols for each molecule are presented according to molecular functions and type of interactions. Prediction legends showed color schemes of shapes and connecting lines

The PDGF signaling pathway appeared to be activated in PeM compared to BPR chickens. PDGFR α and β subunits, and their downstream signal transduction molecules, including JAK, SRC, STAT1 (signal transducer and activator of transcription 1), STAT3, RAS (ras oncogene), PLCγ (phospholipase C, gamma), PKC (protein kinase C), cJUN (cellular Jun protooncogene), cFOS (cellular FBJ murine osteosarcoma viral oncogene homolog), MEKK1 (MAPK/ERK kinase kinase, a.k.a. MAP3K1, mitogen-activated protein kinase kinase kinase 1), MKK4 (mitogen activated protein kinase 4, a.k.a. MAP2K4, mitogen activated protein kinase kinase 4), and JNK1 were upregulated or predicted to be activated (Fig. 3). Since skeletal muscle cell itself does not proliferate and PDGF is known to stimulate the proliferation of muscle satellite cells [24, 25], increased activity of PDGF signaling pathways to STAT1/STAT3 complex [27] and to cJUN/cFOS complex [28] may influence satellite cell formation and its proliferation, resulting in greater muscle cell growth in PeM chickens compared with BPR. Additionally, upregulation of PDGF signaling pathways in PeM muscle may represent a possible subclinical condition of monocyte infiltration of the regenerating muscle tissues, which has been observed as part of wooden breast myopathy in modern commercial broilers [17, 19].

#### Up-regulation of NFE2L2 mediated oxidative stress pathway

**Fig. 4:**
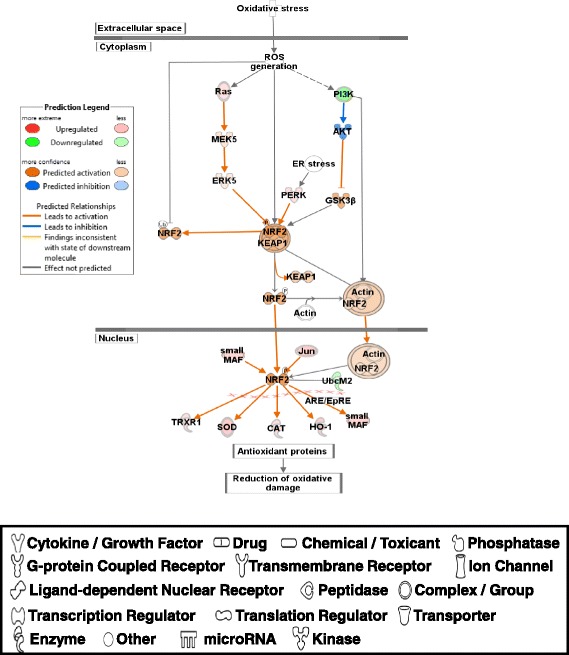
NRF2 mediated oxidative stress pathway. Molecular interaction, symbols, and color schemes are the same as the description in Fig. 3. White symbols indicate neighboring genes that are functionally associated, but not included in the DE gene list

Recently, Zhou et al. [5], reported that with commercial broiler chickens, NFE2L2 (nuclear factor, erythroid 2-like 2; also known as NRF2; Fig. 4) mediated oxidative stress pathway was predicted to be activated in breast muscles in PeM compared to low feed efficiency muscle in commercial broilers. In addition, our recent proteomics results showed that NFE2L2 was also predicted to be activated in the high feed efficiency pedigree male [26]. NFE2L2 as a key transcription factor induces a number of antioxidant genes in response to oxidative stress as well as tissue injury and inflammation [29]. In the present study, NFE2L2 was predicted to be activated in PeM compared to BPR based on the increased expression of downstream targets including TRXR1 (thioredoxin reductase 1 or TXNRD1 in Additional file 2), SOD (superoxide reductase or SOD3 in Additional file 2), CAT (catalase), HO-1 (heme oxygenase 1 or HMOX1 in Additional file 2) and MAF (v-maf avian musculoaponeurotic fibrosarcoma oncogene homolog or MAFA/MAFF in Additional file 2) (Fig. 4). However, the expression of other molecules that respond to NFE2L2 were variable (Table 5). In addition, many other genes [CAT, CCL5 (chemokine C-C motif ligand 5), DUSP1 (dual specificity phosphatase 1), FOS, JUN, MGST1 (microsomal glutathione S-transferase 1), PRDX4 (peroxiredoxin 4), SOD3, STAT3, VCAM1 (vascular cell adhesion molecule 1), and XDH (xanthine dehydrogenase)], along with several antioxidant genes associated with oxidative stress, were upregulated in PeM muscle compared to BPR (Table 6). Earlier biochemical studies for mitochondrial functions and oxidative respirations with breast muscle tissues of high- and low feed efficiency chickens reported lowered ROS levels in high feed efficiency chickens [10, 22], indicating high feed efficiency muscles (or rapidly growing muscles) retain more efficient scavenging mechanisms to reduce ROS levels compared to low feed efficiency muscle. Thus, rather than the possibilities that higher levels of oxidative stress may play a role for PeM, the fact that maintaining low ROS levels caused by higher antioxidant functions is necessary for rapid growth would be a better explanation for the biological functions of the DE genes. Taken together, NFE2L2 mediated antioxidant/detoxifying pathway appears to be more active in muscle of modern PeM broilers compared to BPR chickens.

**Table 5:** DE genes in NFE2L2 mediated oxidative stress

Symbol	Entrez gene name	Fold change	*p*-value	Location	Type (s)
ATM	ATM serine/threonine kinase	−1.31	3.95E-02	Nucleus	kinase
CAT	catalase	1.67	7.26E-05	Cytoplasm	enzyme
DNAJB5	DnaJ (Hsp40) homolog, subfamily B, member 5	1.56	1.61E-02	Cytoplasm	other
DNAJB6	DnaJ (Hsp40) homolog, subfamily B, member 6	1.30	5.86E-03	Nucleus	transcription regulator
DNAJB9	DnaJ (Hsp40) homolog, subfamily B, member 9	−1.45	1.94E-03	Nucleus	other
DNAJC15	DnaJ (Hsp40) homolog, subfamily C, member 15	−1.39	2.04E-02	Cytoplasm	other
DNAJC21	DnaJ (Hsp40) homolog, subfamily C, member 21	−1.97	3.50E-05	Other	other
EIF2AK3	eukaryotic translation initiation factor 2-alpha kinase 3	1.39	1.03E-02	Cytoplasm	kinase
FOS	FBJ murine osteosarcoma viral oncogene homolog	4.66	1.06E-02	Nucleus	transcription regulator
HMOX1	heme oxygenase 1	2.53	7.47E-04	Cytoplasm	enzyme
HRAS	Harvey rat sarcoma viral oncogene homolog	1.43	1.51E-03	Plasma Membrane	enzyme
JUN	jun proto-oncogene	2.45	2.23E-03	Nucleus	transcription regulator
MAP2K3	mitogen-activated protein kinase kinase 3	1.33	1.13E-02	Cytoplasm	kinase
MAP2K6	mitogen-activated protein kinase kinase 6	−1.85	1.31E-02	Cytoplasm	kinase
MGST1	microsomal glutathione S-transferase 1	1.36	3.58E-02	Cytoplasm	enzyme
MRAS	muscle RAS oncogene homolog	1.53	4.77E-02	Plasma Membrane	enzyme
PIK3CA	phosphatidylinositol-4,5-bisphosphate 3-kinase, catalytic subunit alpha	−1.34	1.15E-02	Cytoplasm	kinase
PIK3R1	phosphoinositide-3-kinase, regulatory subunit 1 (alpha)	−2.66	1.05E-03	Cytoplasm	kinase
PRKCB	protein kinase C, beta	2.28	2.15E-03	Cytoplasm	kinase
PRKCD	protein kinase C, delta	1.41	2.50E-02	Cytoplasm	kinase
PRKCH	protein kinase C, eta	−2.95	7.23E-04	Cytoplasm	kinase
SOD3	superoxide dismutase 3, extracellular	2.51	1.48E-02	Extracellular Space	enzyme
TXNRD1	thioredoxin reductase 1	1.74	7.60E-04	Cytoplasm	enzyme

**Table 6:** DE genes in oxidative stress pathway

Symbol	Entrez gene name	Fold change	*p*-value	Location	Type (s)
CAT	catalase	1.67	7.26E-05	Cytoplasm	enzyme
CCL5	chemokine (C-C motif) ligand 5	4.47	3.90E-04	Extracellular Space	cytokine
DUSP1	dual specificity phosphatase 1	2.73	4.81E-03	Nucleus	phosphatase
FOS	FBJ murine osteosarcoma viral oncogene homolog	4.66	1.06E-02	Nucleus	transcription regulator
JUN	jun proto-oncogene	2.45	2.23E-03	Nucleus	transcription regulator
MGST1	microsomal glutathione S-transferase 1	1.36	3.58E-02	Cytoplasm	enzyme
PRDX4	peroxiredoxin 4	1.40	1.35E-02	Cytoplasm	enzyme
SOD3	superoxide dismutase 3, extracellular	2.51	1.48E-02	Extracellular Space	enzyme
STAT3	signal transducer and activator of transcription 3	1.30	7.34E-04	Nucleus	transcription regulator
VCAM1	vascular cell adhesion molecule 1	1.50	2.49E-02	Plasma Membrane	transmembrane receptor
XDH	xanthine dehydrogenase	3.86	1.78E-04	Cytoplasm	enzyme

### Upstream regulators and network analyses

Upstream regulators and gene network analyses, which represent the intermolecular connections among interacting genes based on functional knowledge inputs, were performed on the DE genes using the IPA program. IPA analyses selected 11 potentially important upstream regulators (p-value <0.05) and 9 meaningful molecular networks (data not shown). Interestingly most networks were centered with the upstream regulators. The upstream regulators centered in the molecular networks, that are possibly important for muscle growth and feed efficiency are presented below and gene information including full gene name and fold change values for focus molecules in each network are listed in Additional file 5.

#### MSTN

**Fig. 5:**
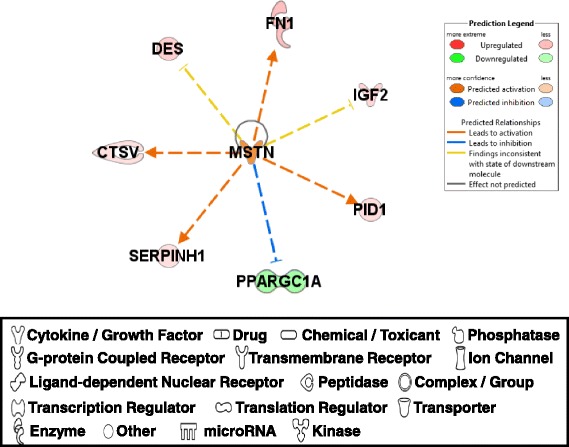
DE genes regulated by MSTN. Molecular interaction, symbols, and color schemes are the same as the description in Fig. 3

The first molecule of interest was MSTN (myostatin), a member of TGF-β family, which is an important negative regulator of skeletal muscle growth [30]. MSTN expression was higher in PeM muscle compared to BPR muscle (Additional file 2). Although the DE of a few downstream target molecules [e.g. DES (desmin) and IGF2 (insulin like growth factor 2)] was indicated as being inconsistent with previous reports [31, 32], the downstream molecules of MSTN including increased expression of FN1 (fibronectin 1), PID1 (phosphotyrosine interaction domain containing 1), SERPINH1 [serpin peptidase inhibitor, clade H (heat shock protein 47), member 1, (collagen binding protein 1)], CTSV (cathepsin V) and decreased expression of PPARGC1A [peroxisome proliferator-activated receptor gamma, coactivator 1 alpha; PGC1α] [31–34] indicated that the functional MSTN activity or expression was enhanced in PeM compared to BPR (Fig. 5). Since PeM chickens grow much faster than BPR, MSTN functions in PeM were expected to be less active than BPR, but the result was opposite. The qPCR of MSTN was consistent with RNAseq result (Table 1) and no single nucleotide polymorphisms (SNP) altering an amino acid residue were found in MSTN cDNA sequences in both PeM and BPR chickens (data not shown). According to a recent RNAseq report comparing a modern pedigree broiler (ROSS) muscle and to that in heritage chicken (Illinois) muscles, MSTN expression was very similar in both chicken lines and showed relatively high expression levels (~30 RPKMs) in both lines, though slightly higher expression was observed in pedigree broiler muscles than heritage chicken muscles [7]. Similarly, the MSTN expression in this study showed relatively abundant expression levels in both BPR (range of 380 – 1000 raw read counts) and in PeM (range of 1000 – 2100 raw read counts) breast muscles (data not shown). Thus, assuming no mutations were involved in the MSTN gene in both lines, high expression levels of MSTN could be hypothesized to prevent extreme muscle growth in both PeM and BPR. The regulatory factors that modulate the function of TGF-β family including MSTN, secreted protein follistatin (FST), together with its associated proteins (follistatin like 1, FSTL1 and follistatin like 3, FSTL3) are known as potent antagonists of myostatin that take advantage of its ability to hinder access to signaling receptors on skeletal muscle [35, 36]. The DE genes in this study showed that FST and FSTL1 expression were upregulated by 3.86 and 1.73 fold changes, respectively, in PeM compared with BPR muscles (Additional file 2), while FSTL3 expression was not detected. Function of abundantly expressed MSTN appears to be attenuated by the autocrine or paracrine effects of upregulated FST and its associated FSTL1 in PeM muscles compared to BPR muscles, resulting in the greater muscle growth in PeM chickens. Therefore, the MSTN functions on the muscle growth may be mediated by the interactions between MSTN and its regulatory factors (e.g. FST and FSTLs), not by the differential expression of MSTN alone. The functional roles of MSTN, its downstream effectors, and FST regulation in PeM compared with BPR need to be investigated more in future studies.

#### PRKAG3

**Fig. 6:**
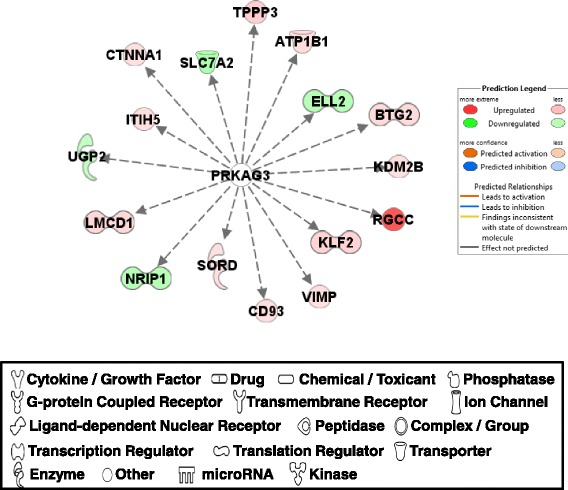
DE genes regulated by PRKAG3. Molecular interaction, symbols, and color schemes are the same as the description in Fig. 3

The regulations centered with PRKAG3 (protein kinase, AMP-activated, gamma 3 non-catalytic subunit) were closely associated with functions in organismal injury/abnormalities, and cellular assembly/organization (Fig. 6). Although there was no expression difference observed between PeM and BPR, mRNA sequence analysis of PRKAG3 in BPR muscle revealed an allele specific expression at nucleotide position (c.943G > A) that induced a nonsynonymous amino acid change (p. V315M) in 5 out of 6 BPR chickens; however, this was not observed in PeM sequence results (data not shown). Three and two BPR samples showed 50% (heterozygous GA genotype) and 100% mutations (homozygous AA genotype), respectively, for PRKAG3. Therefore, DE genes associated with PRKAG3 appeared to be caused by the differential protein/kinase function. Expression for TPPP3 (tubulin polymerization-promoting protein family member 3), ATP1B1 (ATPase, Na+/K+ transporting, beta 1 polypeptide), BTG2 (BTG family, member 2), KDM2B (lysine-specific demethylase 2B), RGCC (regulator of cell cycle), KLF2 (Kruppel-like factor 2), VIMP (VCP-interacting membrane protein), CD93 (CD93 molecule), SORD (sorbitol dehydrogenase), LMCD1 (LIM and cysteine-rich domains 1), ITIH5 (inter-alpha-trypsin inhibitor heavy chain family, member 5), and CTNNA1 [catenin (cadherin-associated protein), alpha 1, 102 kDa] were increased in PeM compared to BPR, while expression of SLC7A2 [solute carrier family 7 (cationic amino acid transporter, y + system), member 2], ELL2 (elongation factor, RNA polymerase II, 2), NRIP1 (nuclear receptor interacting protein 1) and UGP2 (UDP-glucose pyrophosphorylase 2) were decreased by PRKAG3 in PeM. All of these downstream molecules of PRKAG3 are involved in either a) glucose metabolism (SORD [37], UGP2 [38], VIMP [39]), b) cytoskeletal formation (TPPP3 [40], CD93 [41], CTNNA1 [42]), c) cell cycle regulation (BTG2 [43], CD93 [41], RGCC [44]), d) transcription (ELL2 [45], KLF2 [46], LMCD1 [47]), e) transporters in cell/mitochondrial membranes (ATP1B1 [48], SLC7A2 [49]), f) hormone dependent nuclear receptor (NRIP1 [50]), g) ubiquitination (KDM2B [51], BTG2 [43]), or h) inflammatory response (VIMP [39]). The PRKAG3 associated interactions were derived from a list of DE genes identified by microarray assay that was performed with skeletal muscle tissues of PRKAG3 transgenic mice including missense point mutation and knock-out genotypes [52]. PRKAG3 is the muscle-specific predominant γ isoform of adenosine monophosphate (AMP)-activated protein kinase (AMPK) and has been shown to play an important role in glucose uptake, glycogen synthesis, and fat oxidation in white skeletal muscle (fast twitch fiber) found in pig and mouse models carrying a mutation of the gene [53–56]. Differential expression of various downstream effector molecules of PRKAG3 suggests that muscle specific AMPK pathway may play important roles in metabolism of glucose and lipids with additional cellular functions in the muscle growth, regeneration of the degenerated muscle tissues, differential muscle fiber composition, and feed efficiency phenotypic expression in chicken. This concept is supported by the fact that the most relevant molecular and cellular function identified by IPA analysis was carbohydrate metabolism that involved with 16 molecules (Table 4). AMPK has been known to play important roles to regulate feed efficiency related to mitochondrial functions in chicken muscle [2–4]. AMPK functions in monitoring cell energy status and adjusts ATP production to meet cellular needs. The AMPK is stimulated by increased AMP to ATP ratio and enhances energy production by stimulating mitochondrial biogenesis.

#### LIPE

**Fig. 7:**
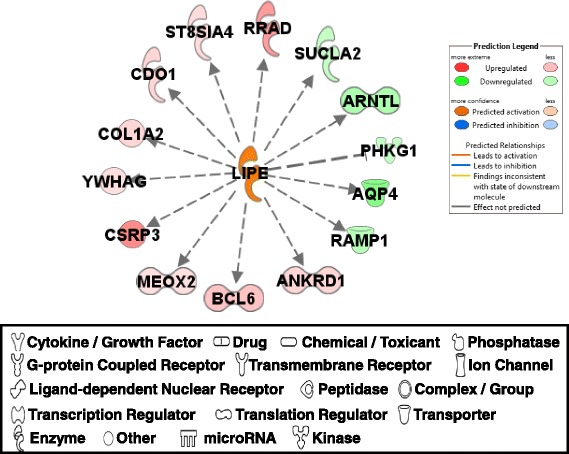
DE genes regulated by LIPE. Molecular interaction, symbols, and color schemes are the same as the description in Fig. 3

The regulation of LIPE (lipase E, hormone sensitive), which is predicted to be activated in PeM compared to BPR, is involved in cell morphology, connective tissue development/function, and tissue morphology (Fig. 7). LIPE associated regulation was derived from an earlier report which identified DE genes and proteins in soleus muscle tissues of LIPE null mice compared with wild-type littermates using microarray and two dimensional gel electrophoresis based mass-spectrometry proteomics assays [57]. Expressions for RRAD (Ras-related associated with diabetes), ST8SIA4 (ST8 alpha-N-acetyl-neuraminide alpha-2,8-sialyltransferase 4), CDO1 (cysteine dioxygenase type 1), COL1A2 (collagen, type I, alpha 2), YWHAG (tyrosine 3-monooxygenase/tryptophan 5-monooxygenase activation protein, gamma), CSRP3 [cysteine and glycine-rich protein 3 (cardiac LIM protein)], MEOX2 (mesenchyme homeobox 2), BCL6 (B-cell CLL/lymphoma 6), and ANKRD1 [ankyrin repeat domain 1 (cardiac muscle)] were up-regulated in PeM compared to BPR, while expression of RAMP1 [receptor (G protein-coupled) activity modifying protein 1], AQP1 (aquaporin 4), PHKG1 [phosphorylase kinase, gamma 1 (muscle)], ARNTL (aryl hydrocarbon receptor nuclear translocator-like) and SUCLA2 (succinate-CoA ligase, ADP-forming, beta subunit) were downregulated. Hormone-sensitive lipase E is a key enzyme in fatty acid mobilization in adipocytes and muscle LIPE has been shown to be activated by adrenaline-mediated protein kinase A phosphorylation in mammals [58] as well as by a contraction-induced mechanism, which is independent of protein kinase A [59]. Although LIPE may have critical functions in mammalian muscle, to date, the avian LIPE gene has not been identified in the genome, transcriptome or proteome. Previously, only a partial 86 kDa protein for the LIPE enzyme was purified from chicken adipose tissue and lipase activity was confirmed [60]. Since mammalian LIPE genes showed high sequence similarity, we tried to identify muscle LIPE gene expression in chicken based on mammalian LIPE gene information but this was unfortunately not successful (data not shown). Therefore, detailed functional discussion on avian muscle LIPE will have to wait until the avian LIPE gene is identified.

#### Androgen receptor (AR)

**Fig. 8:**
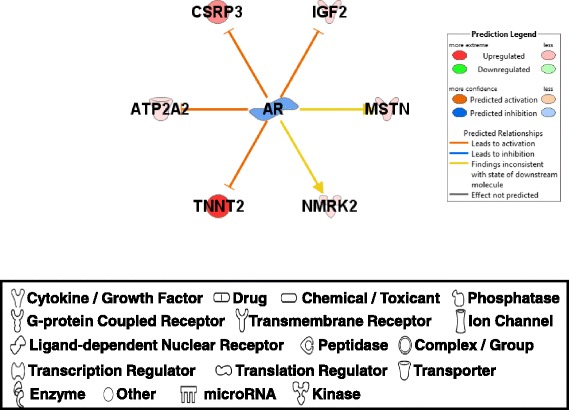
DE genes regulated by androgen receptor (AR). Molecular interaction, symbols, and color schemes are the same as the description in Fig. 3

A steroid hormone receptor, AR (androgen receptor) was predicted to be inhibited in PeM muscle (Fig. 8). AR expression observed from the re-analysis of muscle transcriptome on Ross and Illinois chickens showed a significant downregulation (−2.0 fold, *p*-value = 0.0003) in Ross chicken muscle at 21 day post hatch compared to Illinois chicken [7]. These results support the inhibition of AR functions in modern broiler muscle tissues compared to unselected chicken breeds. AR is a ligand (androgen)-inducible transcription factor that binds to specific DNA sequences called androgen response elements (AREs) via its DNA-binding domain (DBD) and recruits coactivators that facilitate the transcription of target genes [61]. AR and androgen activate muscle-specific genes encoding structural proteins and myogenic factors [62]. Androgens and AR may activate the estrogen receptor after aromatization [63] and play a role in anabolic action in skeletal muscle through crosstalk with other signaling molecules [64]. Although MSTN and NMRK2 (nicotinamide riboside kinase 2), which are known to be transcriptionally activated by AR, were not consistent with earlier findings [65, 66], expression of IGF2, CSRP3 [cysteine and glycine-rich protein 3 (cardiac LIM protein)], ATP2A2 (ATPase, Ca++ transporting, cardiac muscle, slow twitch 2), and TNNT2 [troponin T type 2 (cardiac)] were upregulated in AR receptor knock-out mice [65, 66], suggesting that increased expression of these genes in PeM muscle may be caused by the inhibition of AR functions compared to BPR. Predicted inhibition of AR indicated that steroid receptor- mediated transcriptions were down-regulated in modern broiler PeM compared to progenitor BPR muscles.

### Summary and conclusion

In this study, we have demonstrated DE genes in breast muscle of modern PeM (rapid growth, high feed efficiency) broiler breeder compared to BPR unselected foundational breed (slow growth, low feed efficacy). Thousands of DE genes involved a variety of cellular and physiological functions in muscle growth and energy metabolism. In addition, many differences reflecting differential muscle fiber composition were characterized in the two chicken breeds. The highly upregulated genes in PeM may represent phenotypes of subclinical myopathy commonly observed in the commercial broiler breast tissue, that can lead to muscle hardening, named as woody breast. Importantly, this study suggests that the fiber composition of avian breast muscle is not consistent with mammalian white muscle in regard to differential mitochondrial content and function. The results of this study provide insights into the genetic alterations accumulated in modern broiler breeder line during selective breeding conducted over several decades.

## Additional files

Additional file 1: Primers used for qRT-PCR. The first column indicates primer names and the second column shows sequences for the forward and reverse primers. (XLSX 9 kb)
Additional file 2: List of entire 2464 DE genes in the comparison of PeM and BPR. (XLSX 141 kb)
Additional file 3: Figure S1. The canonical pathway of oxidative phosphorylation. The differentially expressed genes in breast muscle associated with the electron transport chain on (Complex I, II, III, IV, and V) that were downregulated (outlined in green) in the PeM. Pumping of hydrogen ions (H+) creates a proton motive force between the inner (IMM) and outer (OMM) mitochondrial membranes that is used to drive ATP synthesis. (PPTX 254 kb)
Additional file 4: List of DE genes in TCA cycles and oxidative phosphorylation. Gene symbols, full gene name and fold change values were displayed. (XLSX 11 kb)
Additional file 5: List of focus molecules in gene networks and upstream analysis. Gene symbols, full gene name and fold change values were displayed for the illustrations of network and upstream analyses. (XLSX 14 kb)

## Abbreviations

ACE: Angiotensin I converting enzyme
AGTR1: Angiotensin II type 1 receptor
AMP: Adenosine monophosphate
AMPK: Adenosine monophosphate-activated protein kinase
ANGPTL5: Angiopoietin-like 5
ANKRD1: Ankyrin repeat domain 1
AQP1: Aquaporin 4
AR: Androgen receptor
ARE: Androgen response elements
ARNTL: Aryl hydrocarbon receptor nuclear translocator-like
ATP: Adenosine triphosphate
ATP1B1: ATPase Na+/K+ transporting beta 1 polypeptide
ATP2A2: ATPase Ca++ transporting cardiac muscle slow twitch 2
BCL6: B-cell CLL/lymphoma 6
bp: base pair
BPR: Barred Plymouth Rock
BTG2: BTG family member 2
CA3: Carbonic anhydrase 3
CAT: Catalase
CCL5: Chemokine C-C motif ligand 5
CD9: CD9 molecule
CD93: CD93 molecule
cDNA: Complementary DNA
CDO1: Cysteine dioxygenase type 1
cFOS: Cellular FBJ murine osteosarcoma viral oncogene homolog
CGT: UDP glycosyltransferase 8
cJUN: Cellular Jun protooncogene
CKMT1A: Creatine kinase mitochondrial 1A
COL1A2: Collagen type I alpha 2
CSRP3: Cysteine and glycine-rich protein 3
Ct: cycle threshold
CTNNA1: Catenin alpha 1
CTSV: Cathepsin V
DBD: DNA-binding domain
DE: Differentially expressed
DES: Desmin
DUSP1: Dual specificity phosphatase 1
ELL2: Elongation factor 2 RNA polymerase II
EMBL: The European Molecular Biology Laboratory
FABP4: Fatty acid binding protein 4
DKK2: Dickkopf WNT signaling pathway inhibitor 2
FDR: False discovery rate
FGB: Fibrinogen beta chain
FHL2: Four and a half LIM domains 2
FN1: Fibronectin 1
FST: Follistatin
FSTL1: Follistatin like 1
FSTL3: Follistatin like 3
GABRD: Gamma-aminobutyric acid (GABA) A receptor delta
GAPDH: Glyceraldehyde 3-phosphate dehydrogenase
GART: Glycinamide ribonucleotide transformylase; Phosphoribosylglycinamide formyltransferase
GEO: Gene Expression Omnibus
GPM6A: Glycoprotein M6A
GSTA3: Glutathione S-transferase alpha 3
HO-1 or HMOX1: Heme oxygenase 1
IACUC: Institutional Animal Care and Use Committee
IGJ: Joining chain of multimeric IgA and IgM
IGF2: Insulin like growth factor 2
IL23R: Interleukin 23 receptor
IPA: Ingenuity Pathway Analysis
ITIH5: Inter-alpha-trypsin inhibitor heavy chain family member 5
JAK: Janus kinase
KDM2B: Lysine-specific demethylase 2B
KL: Klotho
KLF2: Kruppel-like factor 2
KLF9: Kruppel-like factor 9
KLHL38: Kelch-like family member 38
LIPE: Lipase E
LMCD1: LIM and cysteine-rich domains 1
LMOD2: Leiomodin 2
MAF: V-maf avian musculoaponeurotic fibrosarcoma oncogene homolog
MAP2K4: Mitogen activated protein kinase kinase 4
MAP3K1: Mitogen-activated protein kinase kinase kinase 1
MB: Myoglobin
MEKK1: MAPK/ERK kinase kinase
MEOX2: Mesenchyme homeobox 2
MGST1: Microsomal glutathione S-transferase 1
MKK4: Mitogen activated protein kinase 4
MSTN: Myostatin
MUSTN1: Musculoskeletal embryonic nuclear protein 1
MYBPC1: Myosin binding protein C 1 slow type
MYH15: Myosin heavy chain 15
MYOZ2: Myozenin 2
NFE2L2: Nuclear factor, erythroid 2-like 2
NMRK2: Nicotinamide riboside kinase 2
NOV: Nephroblastoma overexpressed
NOXO1: NADPH oxidase organizer 1
NRF2: Nuclear related factor 2
NRIP1: Nuclear receptor interacting protein 1
OXOPHOS: Oxidative phosphorylation
PCSK1: Proprotein convertase subtilisin/kexin type 1
PeM: Pedigree male
PDGF: Platelet derived growth factor
PDGFR: Platelet derived growth factor receptor
PHKG1: Phosphorylase kinase gamma 1
PKC: Protein kinase C
PID1: Phosphotyrosine interaction domain containing 1
PLCγ: Phospholipase C gamma
PLIN1: Perilipin 1
PLN: Phospholamban
PPARGC1A or PGC1α: Peroxisome proliferator-activated receptor gamma coactivator 1 alpha
PPARGC1B: Peroxisome proliferator-activated receptor gamma, coactivator 1 beta
PPDPF: Pancreatic progenitor cell differentiation and proliferation factor
PRDX4: Peroxiredoxin 4
PRKAG3: Protein kinase AMP-activated gamma 3 non-catalytic subunit
PROCA1: Protein interacting with cyclin A1
qPCR: Quantitative reverse transcription polymerase chain reaction
RAMP1: Receptor (G protein-coupled) activity modifying protein 1
RAS: Ras oncogene
RFI: Residual feed intake
RGCC: Regulator of cell cycle
RNAseq: RNA sequencing
RHOF: Ras homolog family member F
ROS: Reactive oxygen species
RPKM: Reads per kilobase per million
RPM: Reads per million
RRAD: Ras-related associated with diabetes
SCN5A: Sodium channel voltage gated type V alpha subunit
SERPINH1: Serpin peptidase inhibitor clade H heat shock protein 47 member 1
SLC38A4: Solute carrier family 38 member 4
SLC7A2: Solute carrier family 7 member 2
SNP: Single nucleotide polymorphisms
SOD3: Superoxide reductase 3
SORD: Sorbitol dehydrogenase
SRC: SRC protooncogene
ST8SIA4: ST8 alpha-N-acetyl-neuraminide alpha-2,8-sialyltransferase 4
STAT1: Signal transducer and activator of transcription 1
STAT3: Signal transducer and activator of transcription 3
SUCLA2: Succinate-CoA ligase ADP-forming beta subunit
TCA: tricarboxylic acid
TGF-β: Transforming growth factor beta
TNC: Tenascin C
TNNI1: Troponin I 1
TNNT2: Troponin T type 2 cardiac
TPM3: Tropomyosin 3
TPPP3: Tubulin polymerization-promoting protein family member 3
TRXR1 or TXNRD1: Thioredoxin reductase 1
UGP2: UDP-glucose pyrophosphorylase 2
VCAM1: Vascular cell adhesion molecule 1
VIMP: VCP-interacting membrane protein
XDH: Xanthine dehydrogenase
YWHAG: Tyrosine 3-monooxygenase/tryptophan 5-monooxygenase activation protein gamma
