# Supplementary figures and images for: RNA sequencing for global gene expression associated with muscle growth in a single male modern broiler line compared to a foundational Barred Plymouth Rock chicken line
Source: BMC Genomics. 2017 Jan 13;18:82. doi: 10.1186/s12864-016-3471-y (PMC5237145; doi:10.1186/s12864-016-3471-y)

## Slide 1
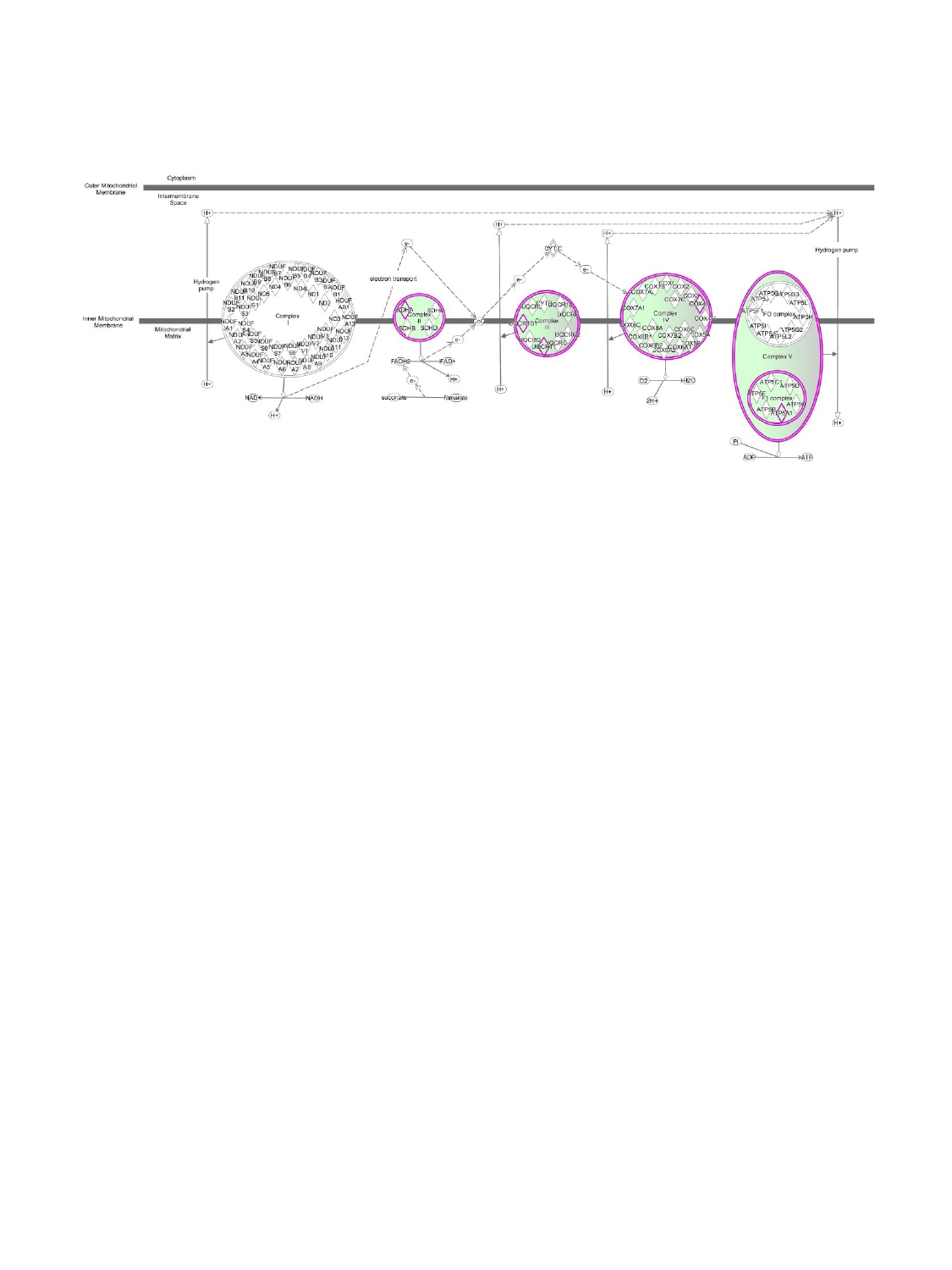

Supplement: Additional file 3: Figure S1. — The canonical pathway of oxidative phosphorylation. The differentially expressed genes in breast muscle associated with the electron transport chain on (Complex I, II, III, IV, and V) that were downregulated (outlined in green) in the PeM. Pumping of hydrogen ions (H+) creates a proton motive force between the inner (IMM) and outer (OMM) mitochondrial membranes that is used to drive ATP synthesis. (PPTX 254 kb) [file 12864_2016_3471_MOESM3_ESM.pptx]
